# Supplementary material for: Medium-sized protein language models perform well at transfer learning on realistic datasets
Source: Sci Rep. 2025 Jul 1;15:21400. doi: 10.1038/s41598-025-05674-x (PMC12217344; doi:10.1038/s41598-025-05674-x)
Supplement: Supplementary file 1 — Supplementary Information. [file 41598_2025_5674_MOESM1_ESM.pdf]

Medium-sized protein language models perform well at transfer learning on realistic datasets

Luiz C. Vieira, Morgan L. Handojo, Claus O. Wilke

May 12, 2025

Supporting Information

Supporting Figures

Supporting Table

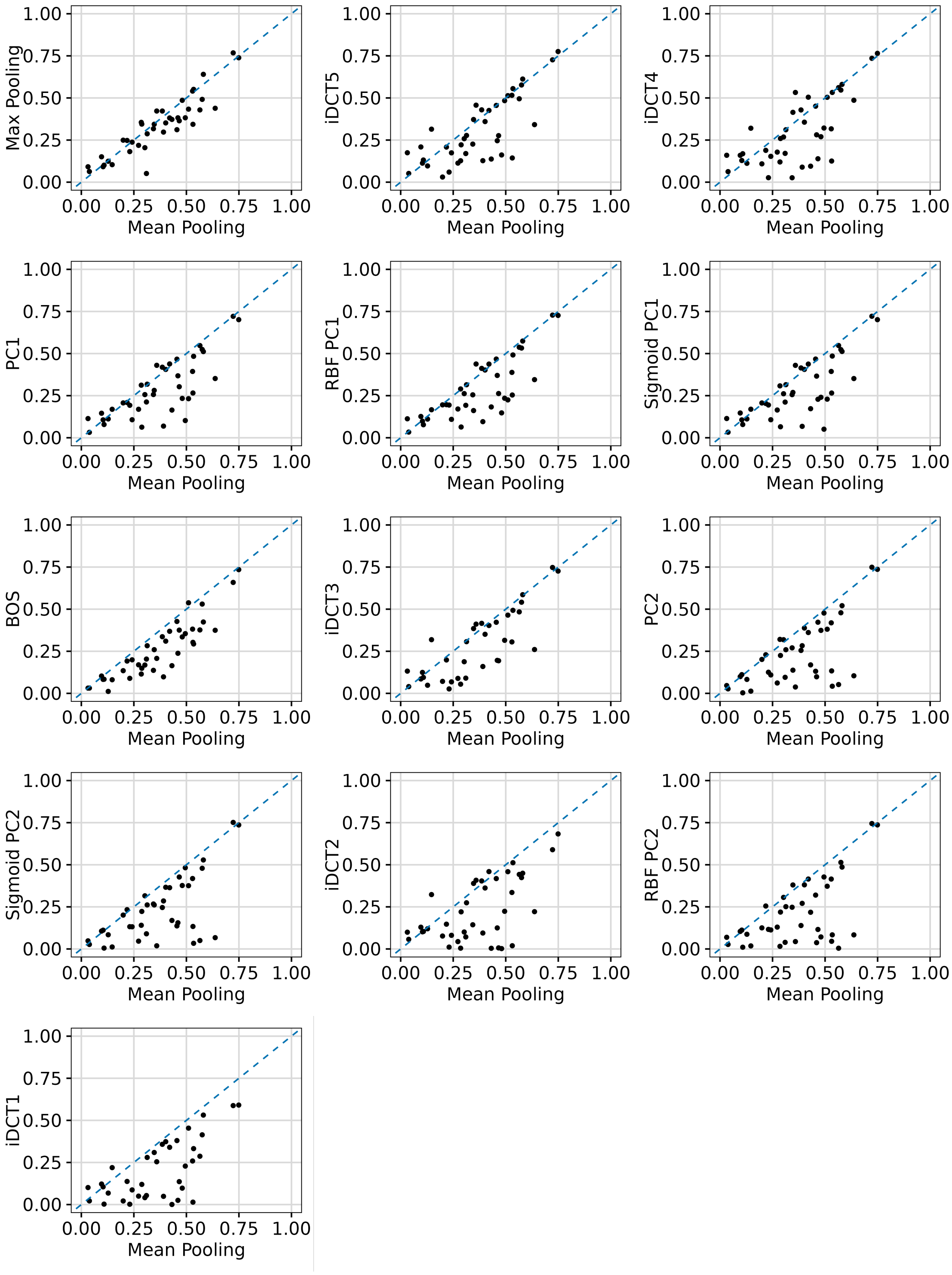

Figure S1: **Comparison of mean embeddings with 13 alternative compression methods on DMS datasets.** In each plot, the x-axis represents the averaged R^2 scores from 5-fold cross-validation for the mean embeddings, while the y-axis shows the averaged R^2 scores from 5-fold cross-validation for one of the other 13 compression methods. Each dot corresponds to one of the 40 DMS datasets analyzed.

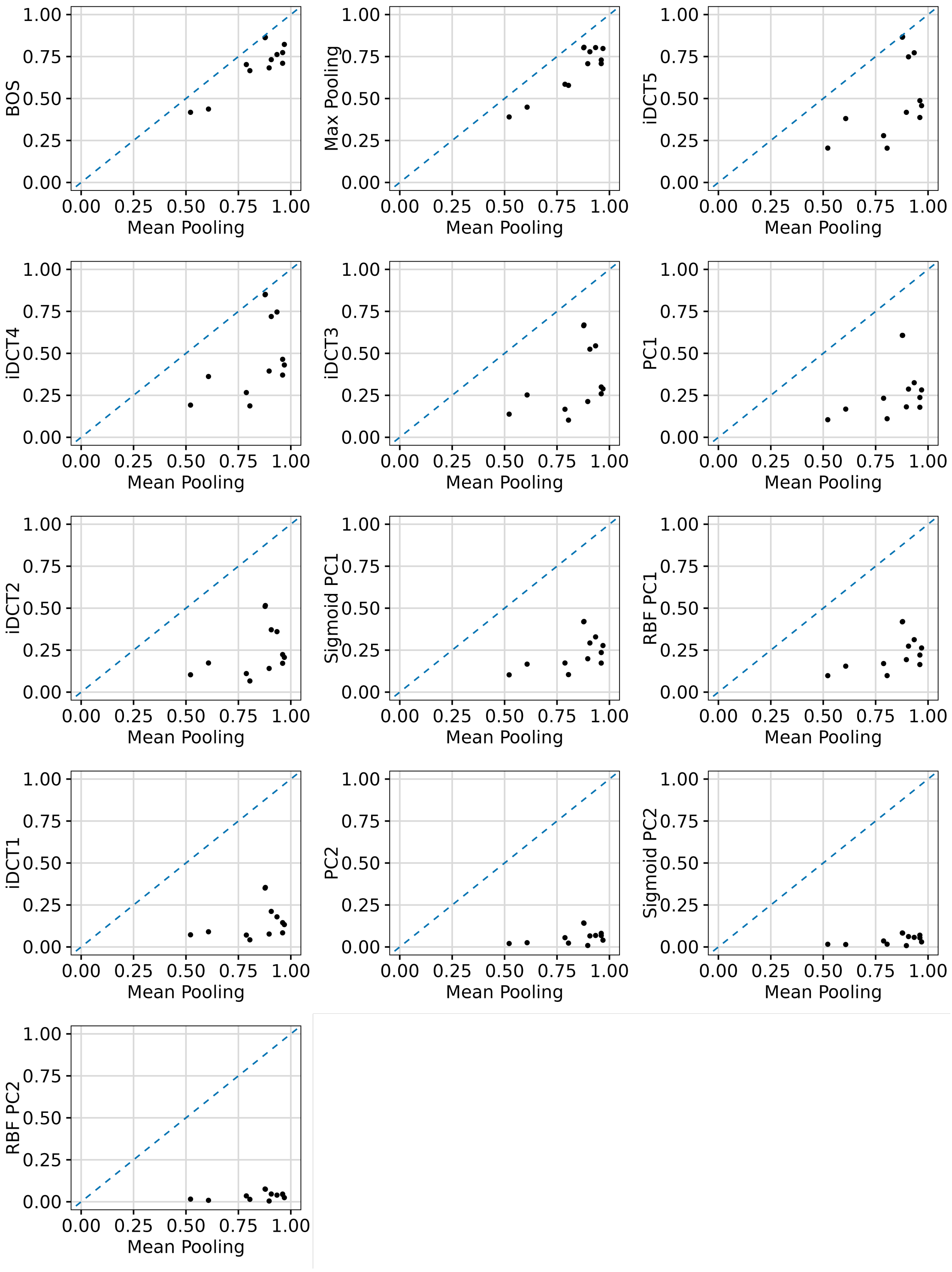

Figure S2: **Comparison of mean embeddings with 13 alternative compression methods on PISCES dataset.** In each plot, the x-axis represents the averaged R^2 scores from 5-fold cross-validation for the mean embeddings, while the y-axis shows the averaged R^2 scores from 5-fold cross-validation for one of the other 13 compression methods. Each dot corresponds to one of the 12 targets analyzed from PISCES dataset (PCPs and Secondary Structure).

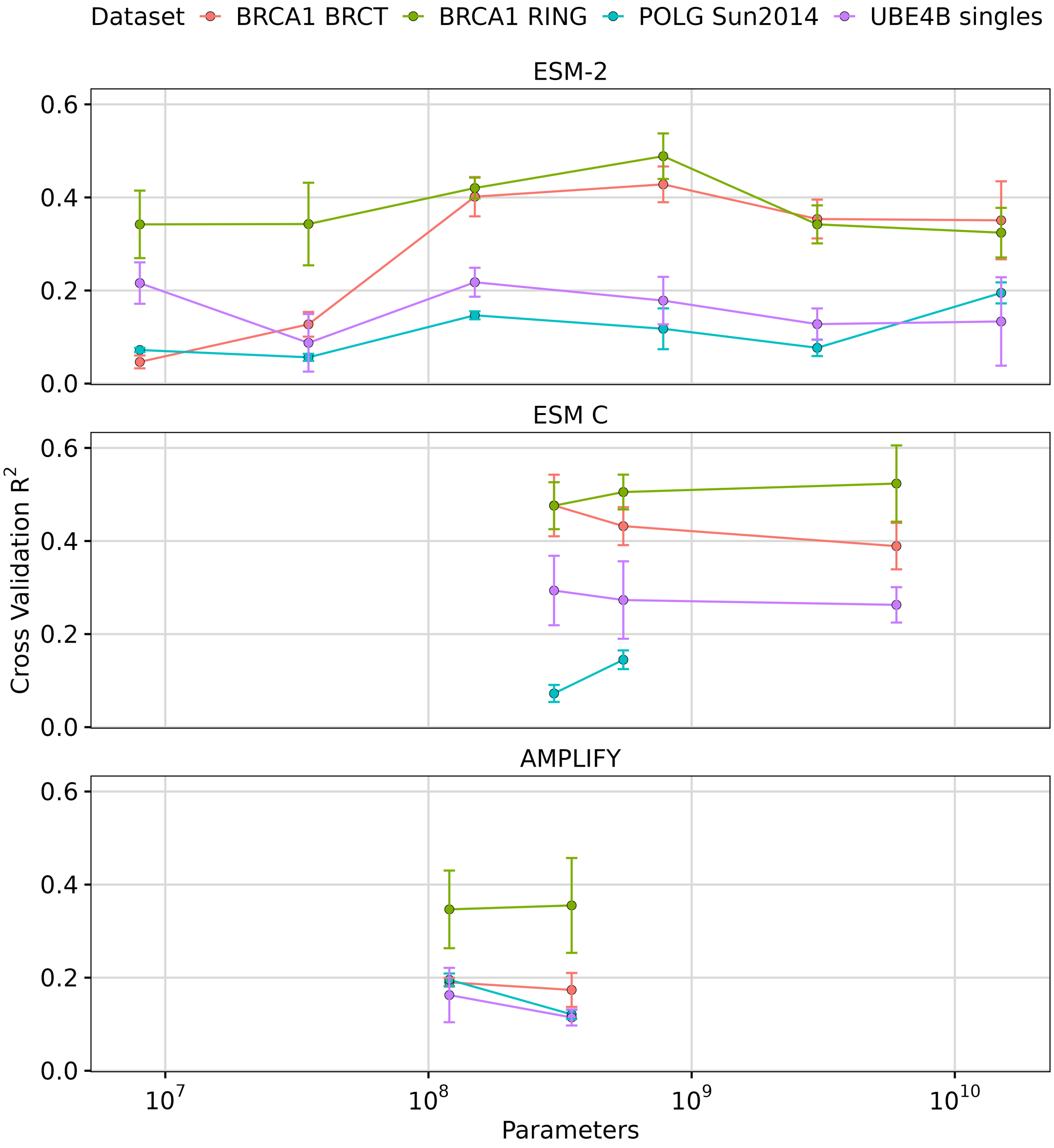

Figure S3: **Performance of pLMs on larger protein sequences.** Prediction scores across 4 different DMS datasets with sequence lengths greater than 1022. The y-axis represents the averaged 5-fold cross-validation R^2 scores, and error bars represent the standard deviation. The x-axis shows the different model sizes. Colors represent individual protein datasets, varying in their lengths: 3033 (POLG Sun2014), 1863 (BRCA1 BRCT), 1863 (BRCA1 RING), 1173 (UBE4B singles).

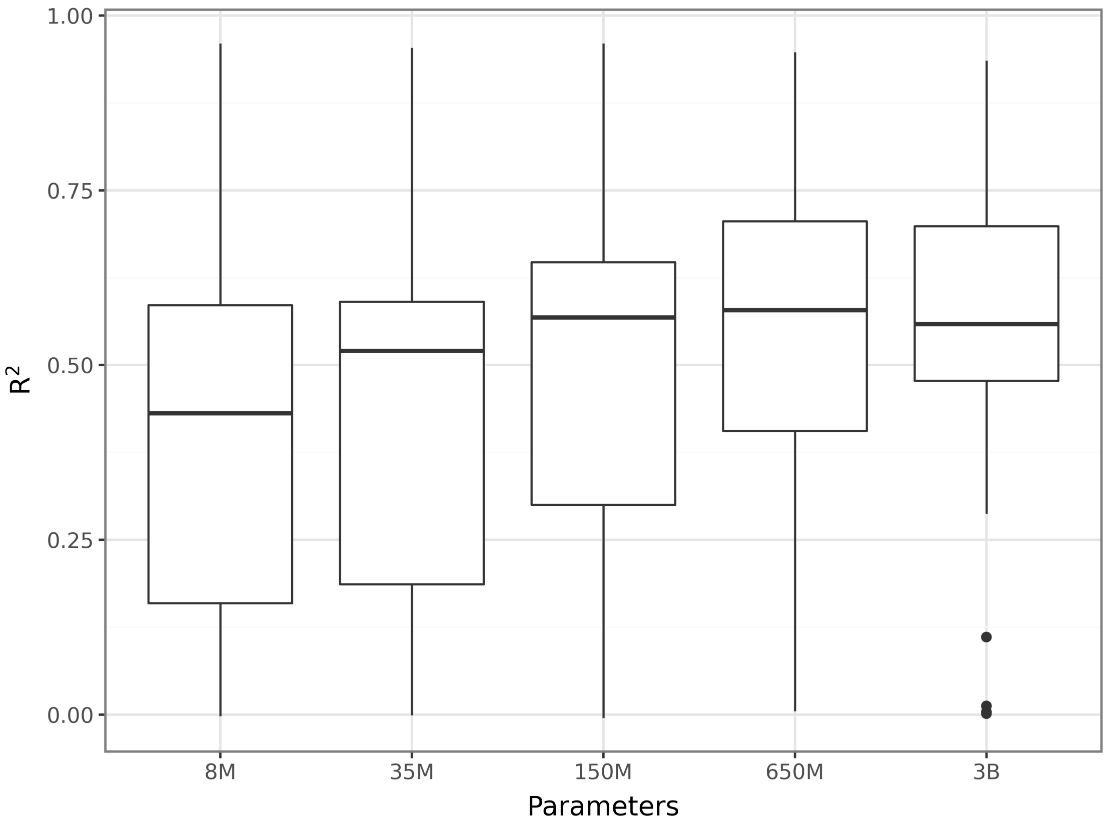

Figure S4: **Impact of model size on fine-tuning ESM-2.** We are plotting the distribution of R^2 scores obtained for 31 different DMS datasets against the number of parameters in the respective ESM-2 model.

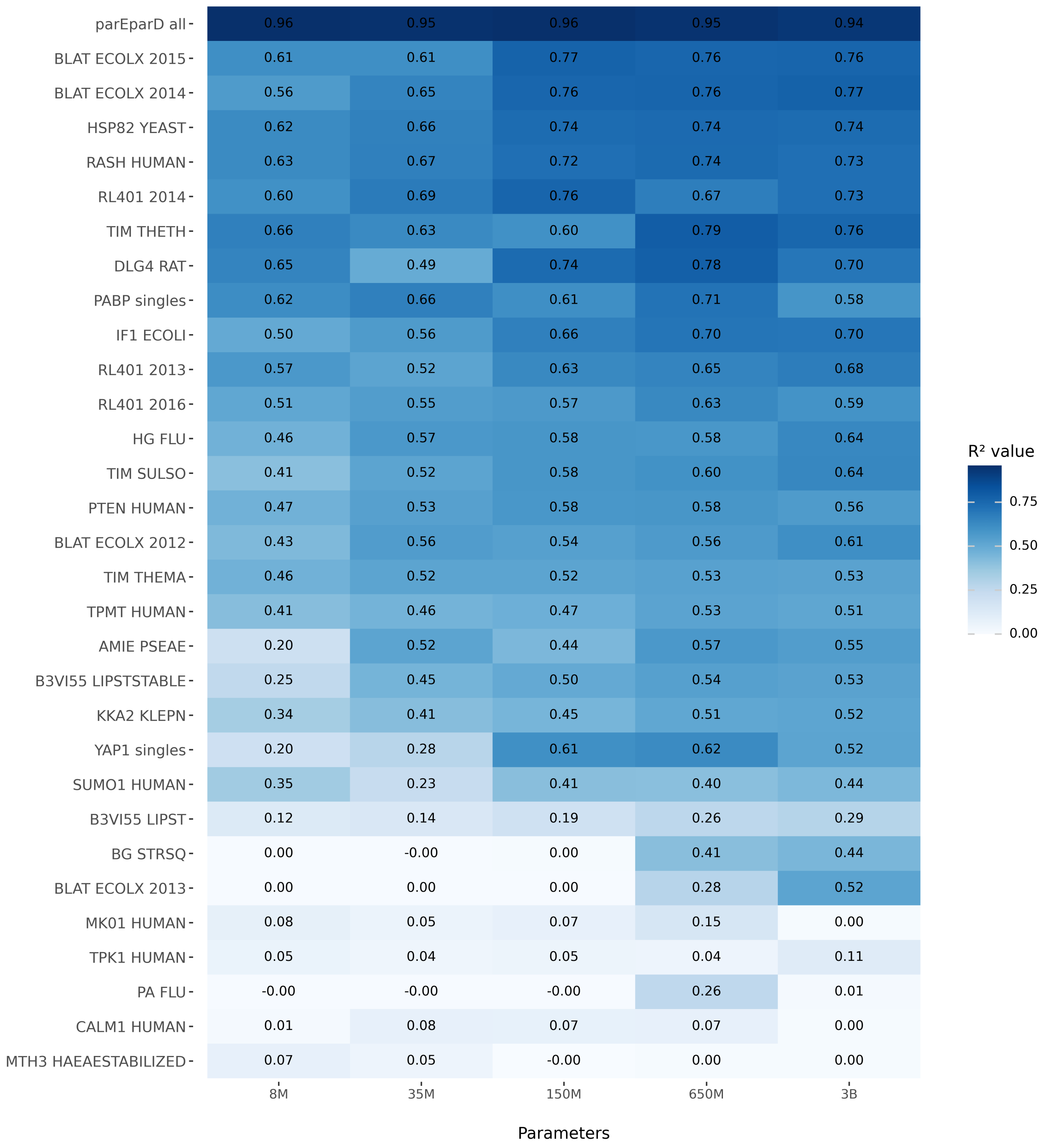

Figure S5: **Impact of model size on fine-tuning ESM-2 per dataset.** We plot the individual R^2 scores obtained for 31 different DMS datasets against the number of parameters in the corresponding ESM-2 models. The R^2 scores are shown in blue, with their corresponding values annotated. Rows are sorted by the average R^2 across models for each dataset.

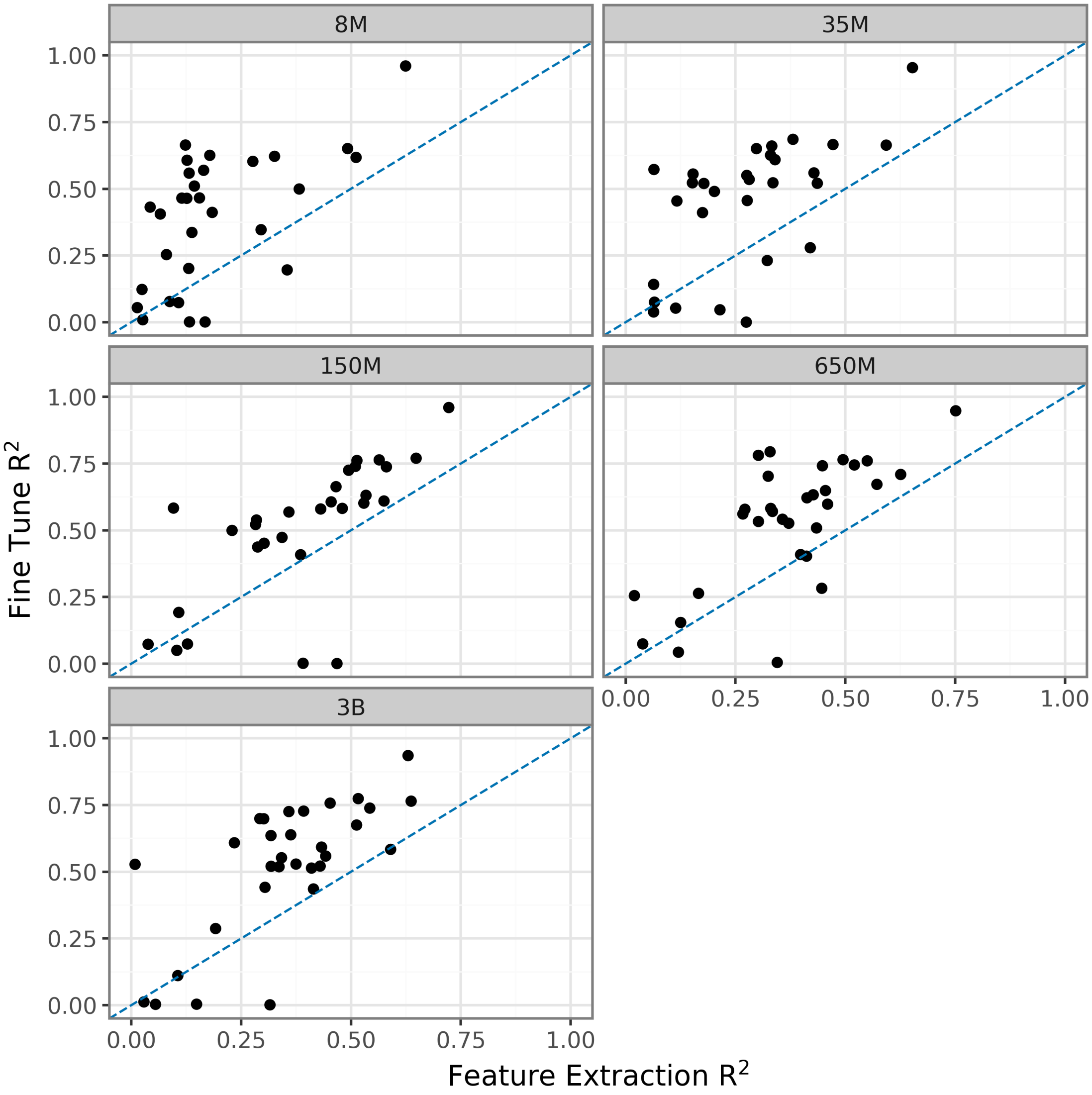

Figure S6: **Comparison between transfer learning strategies.** We are plotting the R^2 scores obtained by fine-tuning ESM-2 versus the R^2 scores obtained by transfer learning via feature extraction and LassoCV, for the same 31 DMS datasets as in Figure S4. The panels correspond to the different ESM-2 model sizes.

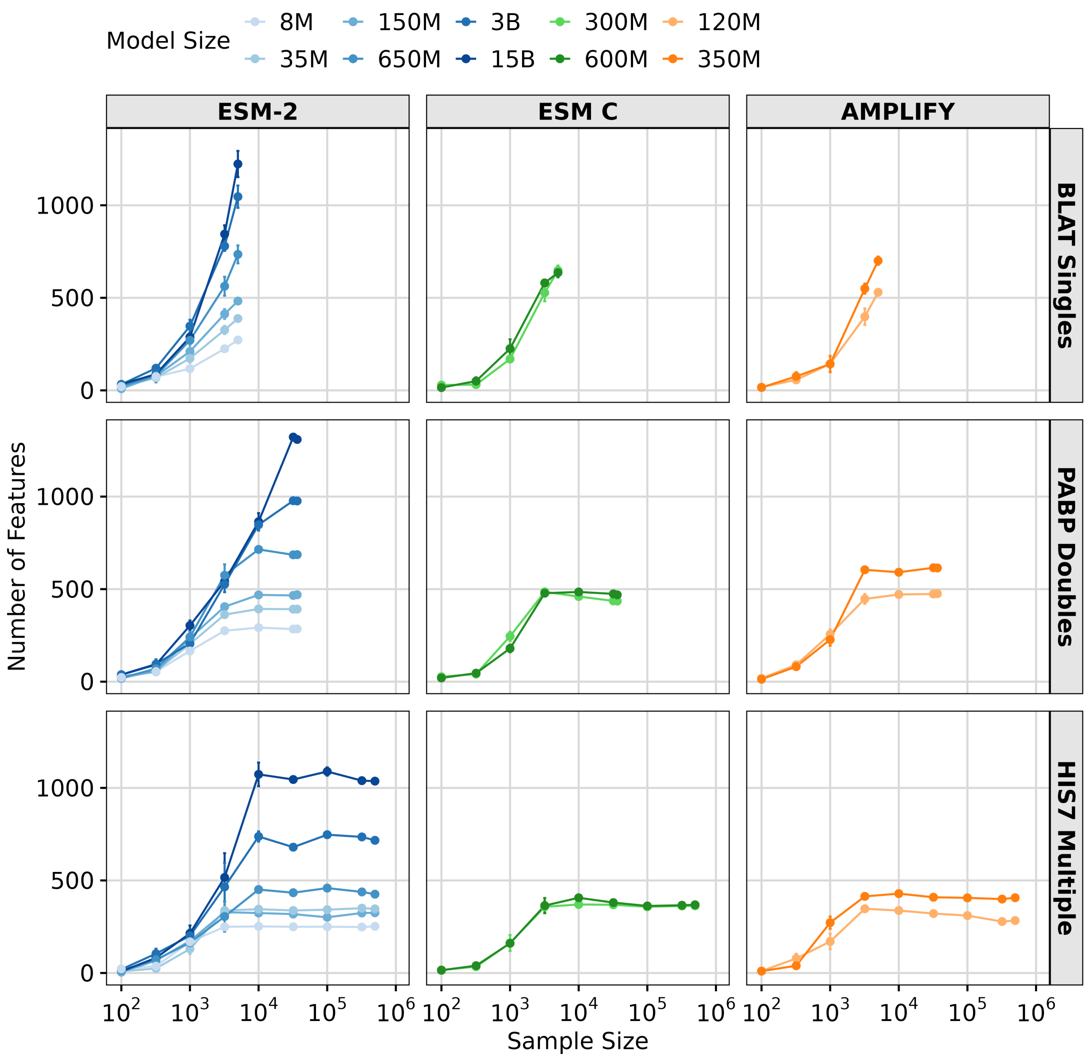

Figure S7: **Number of features boosts performance in protein prediction tasks.** Number of important features identified by LassoCV regression for three DMS datasets with varying mutation complexities: single mutations (BLAT ECOLX - 2015), double mutations (PABP), and multiple mutations (HIS7 YEAST). The y-axis represents the averaged 5-fold cross-validation number of important features (or non-zero coefficients set by Lasso l1 penalization) scores, and error bars represent the standard deviation. ESM models sizes are represented by colored lines: ESM1v 650M (Orange) and ESM-2 8M, 35M, 150M, 650M, 3B, and 15B (Blues)

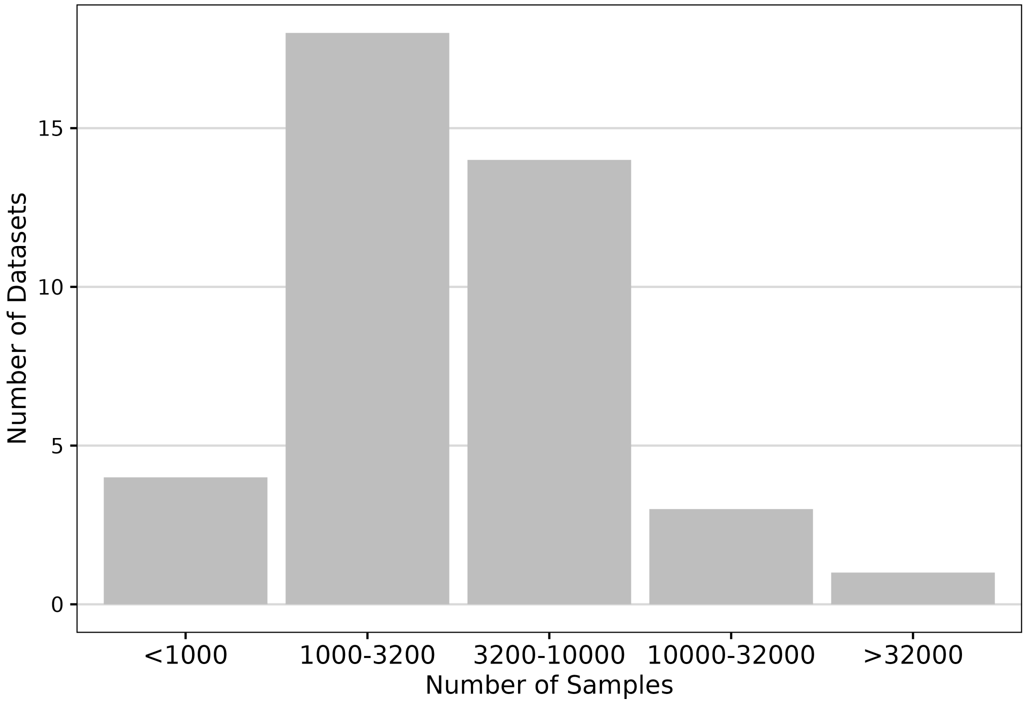

Figure S8: **Sample size distribution in deep mutational scanning (DMS) datasets.**

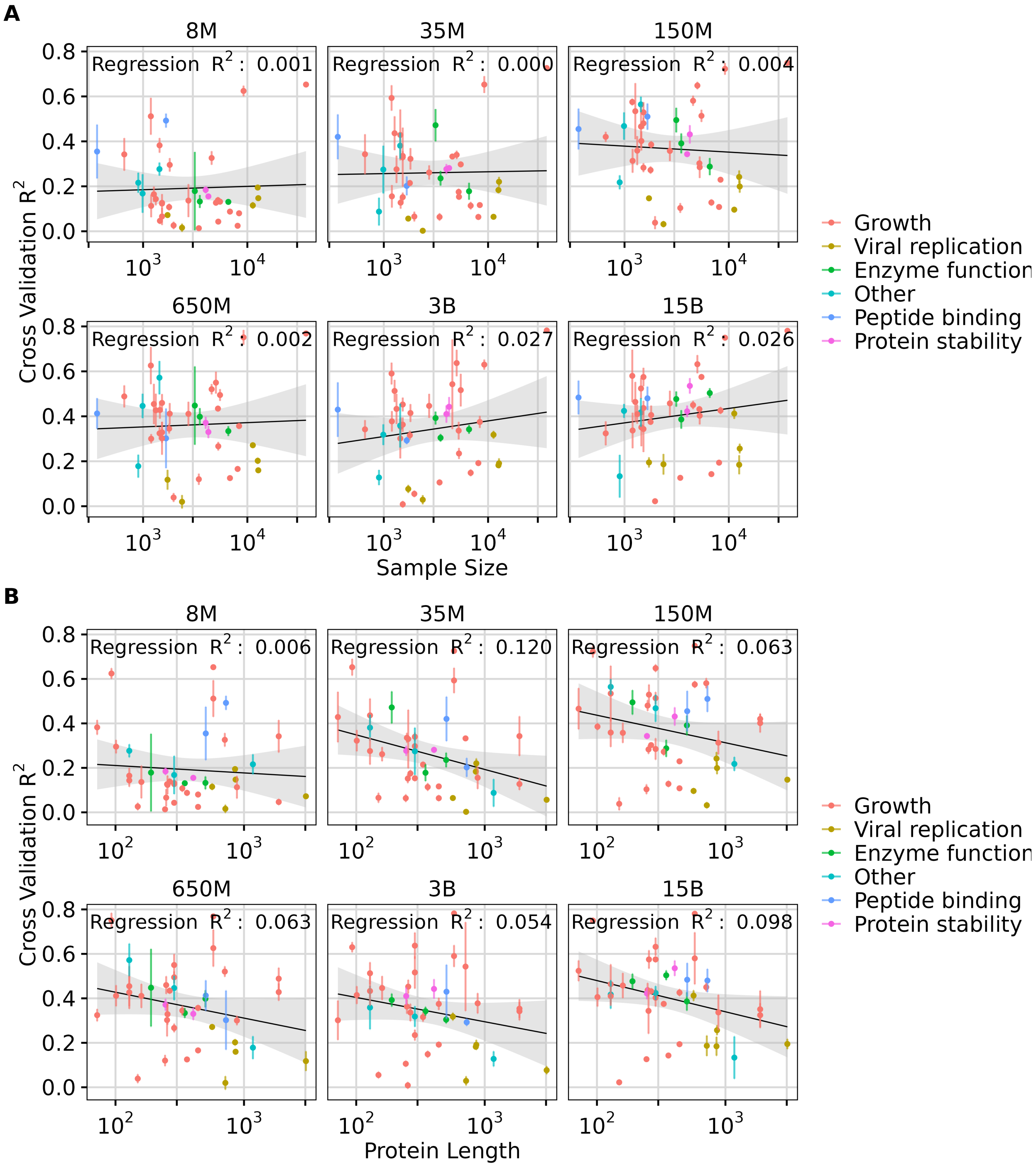

Figure S9: **Effect of sample size and protein length in transfer learning using ESM-2 embeddings.** A) Effect of **sample size** on transfer learning using ESM-2 model embeddings quantified by the regression results (R^2 score). B) Effect of **protein length** on transfer learning using ESM-2 model embeddings quantified by the regression results (R^2 score). Each dot represents a dataset, and its color indicates the corresponding data measurement type. In each subplot, the y-axis represents the averaged 5-fold cross-validation R^2 scores, and error bars represent the standard deviation.

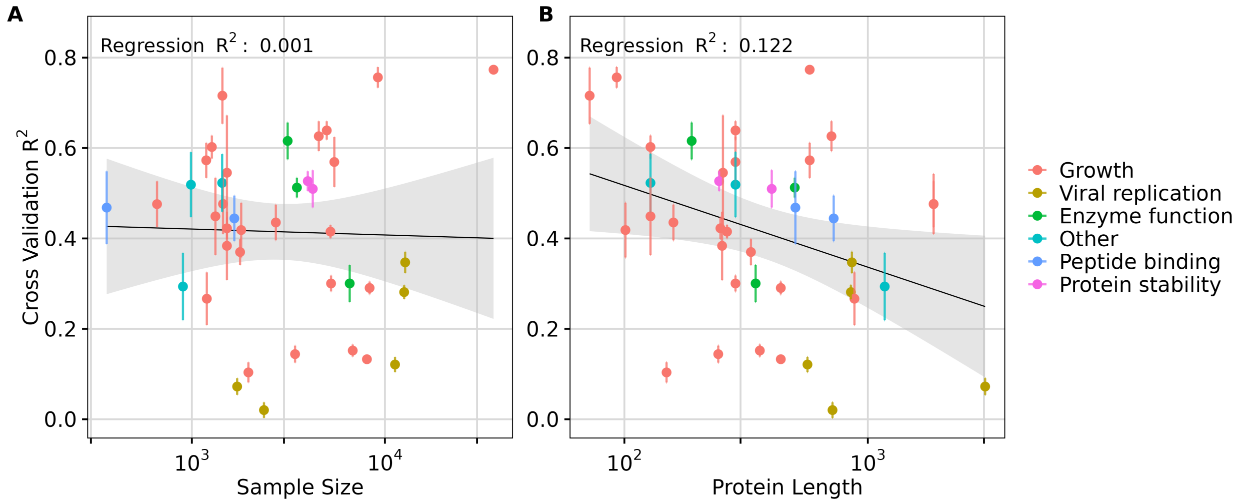

Figure S10: **Effect of sample size and protein length in transfer learning using ESM C 300M embeddings.** A) Effect of **sample size** on transfer learning using ESM C 300M model embeddings quantified by the regression results (R^2 score). B) Effect of **protein length** on transfer learning using ESM C 300M model embeddings quantified by the regression results (R^2 score). Each dot represents a dataset, and its color indicates the corresponding data measurement type. In each subplot, the y-axis represents the averaged 5-fold cross-validation R^2 scores, and error bars represent the standard deviation.

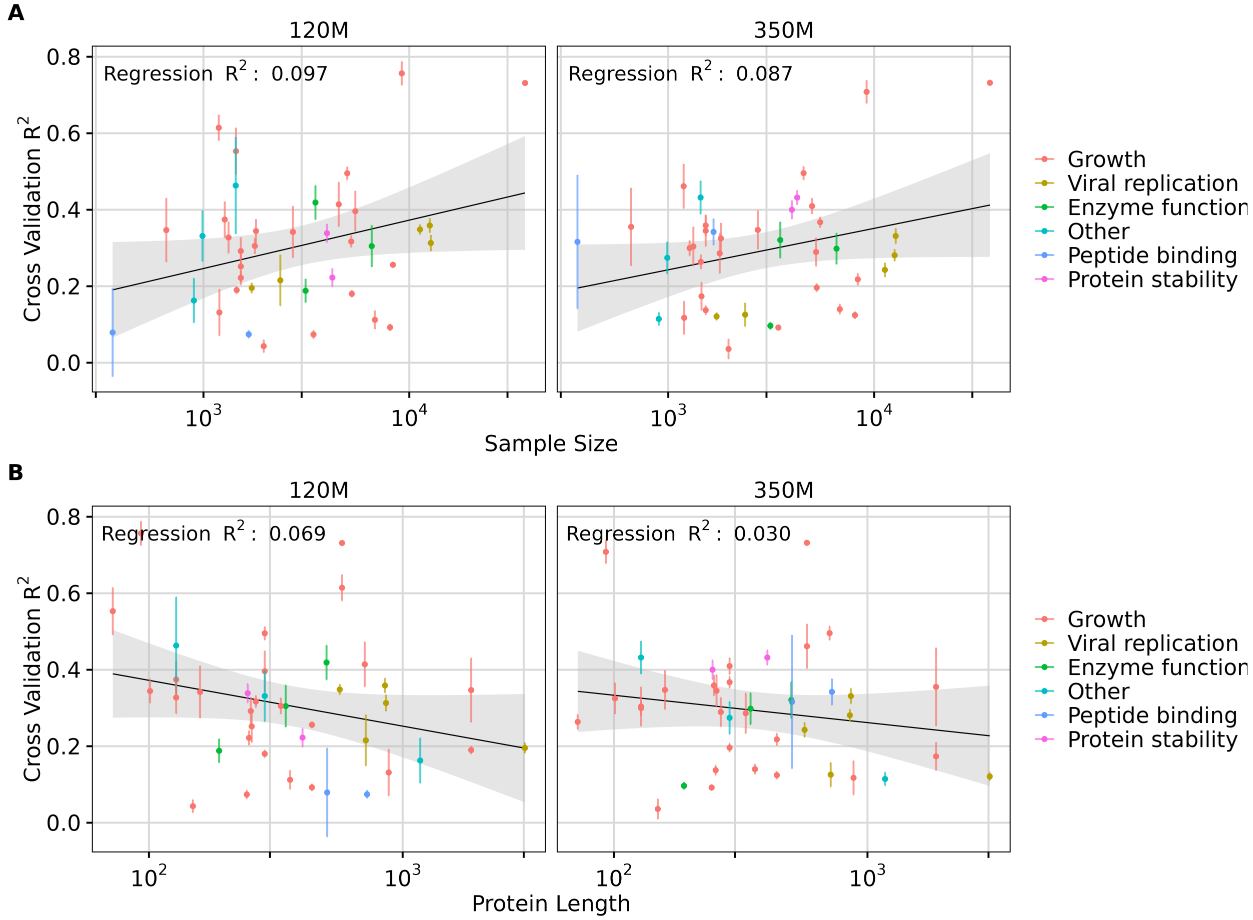

Figure S11: **Effect of sample size and protein length in transfer learning using AMPLIFY embeddings.** A) Effect of **sample size** on transfer learning using AMPLIFY models embeddings quantified by the regression results (R^2 score). B) Effect of **protein length** on transfer learning using AMPLIFY models embeddings quantified by the regression results (R^2 score). Each dot represents a dataset, and its color indicates the corresponding data measurement type. In each subplot, the y-axis represents the averaged 5-fold cross-validation R^2 scores, and error bars represent the standard deviation. The left and right panels represent the two model sizes, 120 million and 350 million parameters, respectively.

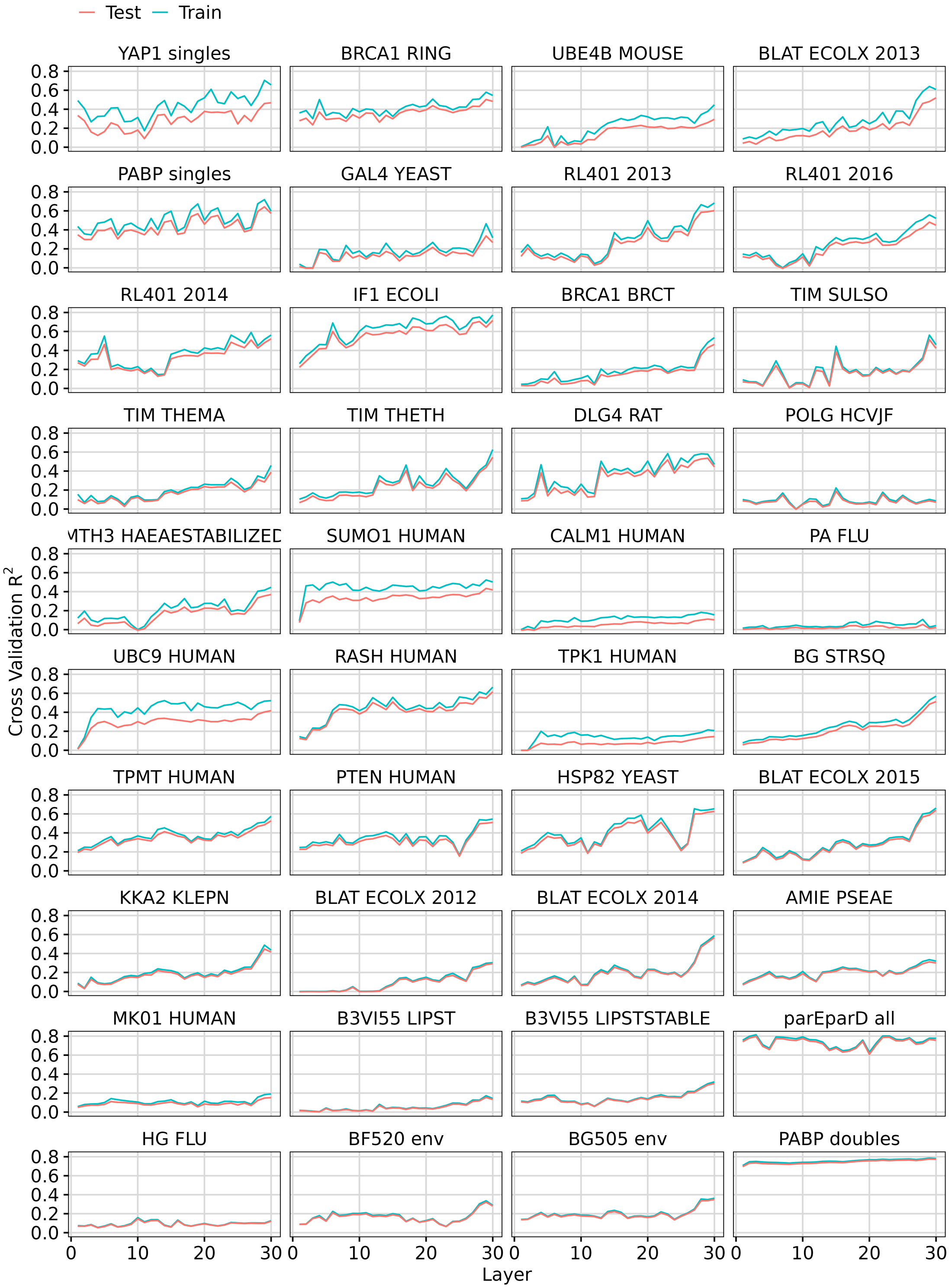

Figure S12: **Performance of Lasso regression using features extracted from different layers of ESMC 300M.** The y-axis represents the average R^2 score from 5-fold cross-validation, and the x-axis indicates the layer number.

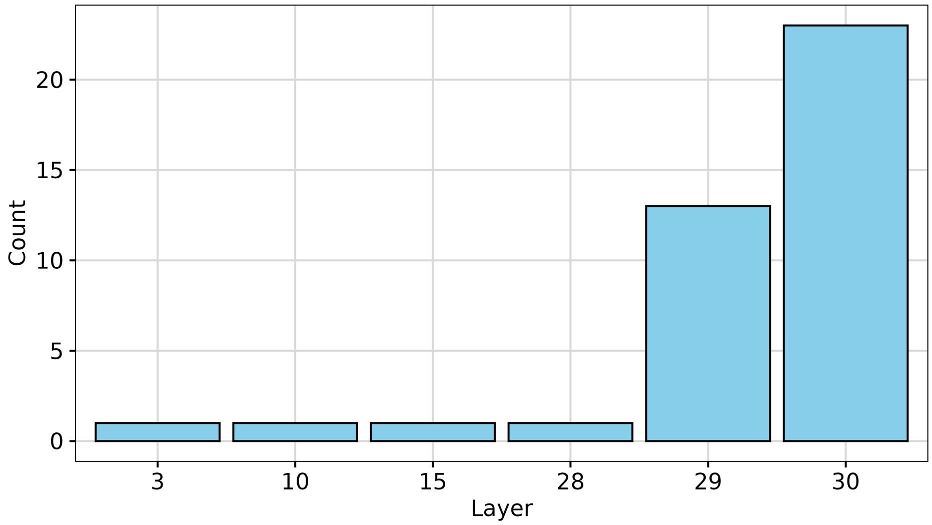

Figure S13: **Cumulative count of datasets for which each layer produced the best performance.** The x-axis represents the layer number used for embedding extraction, and the y-axis shows how many datasets had that layer as the top performer.

Table S1: Target description for PISCES dataset.

**Class**	**Target**	**Definition**
Amino-acid Statistics	Alanine	The fraction of alanine residues present in the protein sequence.
Amino-acid Statistics	Cysteine	The fraction of cysteine residues present in the protein sequence.
Amino-acid Statistics	Leucine	The fraction of leucine residues present in the protein sequence.
Physico-chemical Properties (PCP)	Length	Number of residues in the protein.
Physico-chemical Properties (PCP)	Charge	The theoretical net charge of a protein sequence, based on the Henderson-Hasselbach equation.
Physico-chemical Properties (PCP)	Hydrophobicity	The averaged hydrophobicity values of each residue.
Physico-chemical Properties (PCP)	Instability index	The stability of a protein based on its dipeptide composition.
Physico-chemical Properties (PCP)	Molecular Weight (Kilodaltons)	The sum of the masses of each amino acid.
Physico-chemical Properties (PCP)	Isoelectric point	The isoelectric point (pI), which is the pH at which a particular molecule or surface carries no net electrical charge.
Secondary Structure Frequency (SS)	Extended strand (beta strand)	The fraction residues in extended strand conformation.
Secondary Structure Frequency (SS)	Alpha helix	The fraction of residues in alpha helix conformation.
Secondary Structure Frequency (SS)	Coil	The fraction of residues in coil conformation.
